# Supplementary material for: The Natural Alkaloid Palmatine Selectively Induces Mitophagy and Restores Mitochondrial Function in an Alzheimer’s Disease Mouse Model
Source: Int J Mol Sci. 2023 Nov 20;24(22):16542. doi: 10.3390/ijms242216542 (PMC10671668; doi:10.3390/ijms242216542)
Supplement: Supplementary file 1 [file ijms-24-16542-s001.zip › ijms-2708379-supplementary.pdf]

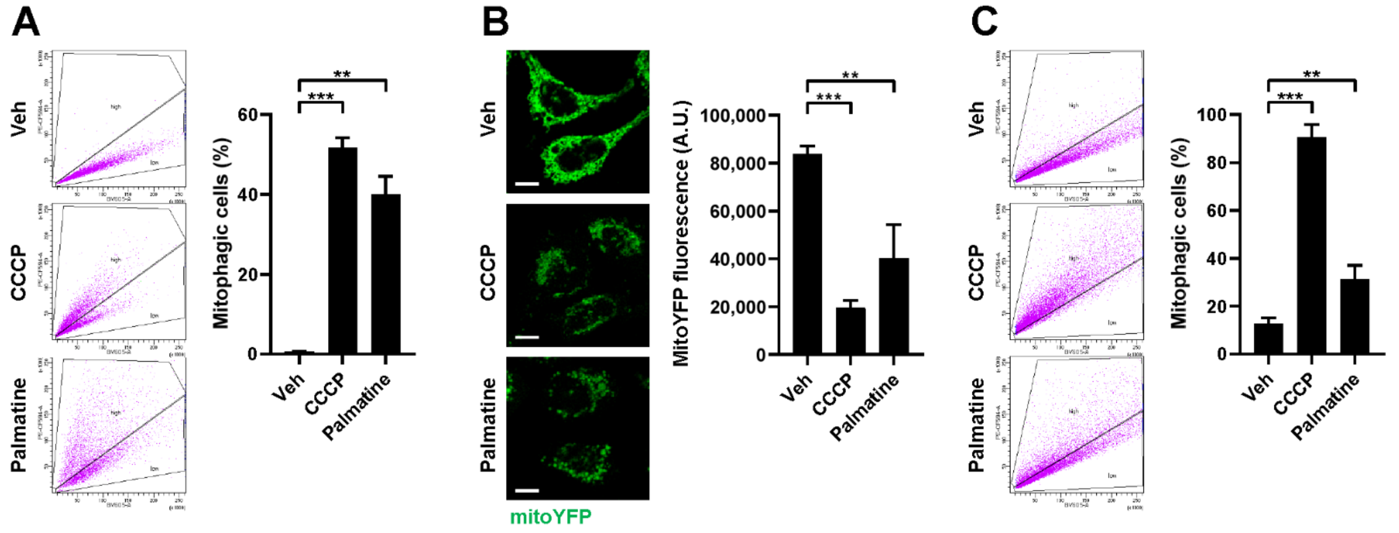

Supplementary Figure S1. Verification of palmatine-induced mitophagy in various human cells.

(A) HeLa-Parkin cells expressing mt-Keima were treated with CCCP (10 μM) or palmatine (400 μM) for 24 h. Mitophagy levels were analyzed by flow cytometry. (B) HeLa-Parkin cells expressing mitoYFP were treated with CCCP (10 μM) or palmatine (400 μM) for 24 h. The fluorescence intensity of mitoYFP was analyzed by confocal microscopy. Scale bar: 10 μm. (C) A549 cells expressing mt-Keima were treated with CCCP (10 μM) or palmatine (400 μM) for 24 h. Mitophagy levels were analyzed by flow cytometry. The results from repeated experiments are shown as the mean ± SD. Significance was determined by one-way ANOVA. ***P* <0.01; ****P* <0.001.
